# A cohort study of dietary iron and heme iron intake and risk of colorectal cancer in women

**DOI:** 10.1038/sj.bjc.6603837

**Published:** 2007-06-05

**Authors:** G C Kabat, A B Miller, M Jain, T E Rohan

**Affiliations:** 1 Department of Epidemiology and Population Health, Albert Einstein College of Medicine, 1300 Morris Park Avenue, Room 1301, NY 10461, USA; 2 Department of Public Health Sciences, University of Toronto, Toronto, Canada

**Keywords:** alcohol, colorectal neoplasms, cohort study, dietary iron, heme iron, hormone replacement therapy, red meat

## Abstract

In a cohort study of 49 654 Canadian women, we assessed the association of colorectal cancer with total iron and heme iron intake, excluding iron supplements. Among women aged 40–59 years, followed for an average of 16.4 years, we identified 617 incident colorectal cancer cases. Data from a food frequency questionnaire administered at baseline were used to calculate red meat intake and intake of total dietary iron, iron from meat, and heme iron. Analyses were carried out for all cases and for the proximal colon, distal colon, and rectum, using Cox proportional hazards models. We found no association of intake of iron, heme iron, or iron from meat with risk of colorectal cancer overall or with any of the subsites, nor was there effect modification by alcohol consumption or hormonal replacement therapy.

Free iron is a pro-oxidant, and may contribute to colorectal carcinogenesis by promoting free radical production and lipid peroxidation (Nelson, 1992; Toyokuni, 1996; Huang, 2003). Epidemiologic studies examining the association of iron intake and markers of body iron stores with risk of colorectal cancer or colorectal polyps have yielded some evidence of positive associations (Knekt *et al*, 1994; Bird *et al*, 1996; Wurzelmann *et al*, 1996; Tseng *et al*, 1997; Kato *et al*, 1999). In addition, epidemiologic studies have shown a modest association between red meat intake, the major source of dietary iron, and risk of colon and colorectal cancer (Norat and Riboli, 2001; Larsson and Wolk, 2006). However, few studies have examined the association of intake of heme iron, derived principally from red meat, with colorectal cancer risk. Heme iron, which has greater bioavailability compared to non-heme iron, has been shown experimentally to have cytotoxic and hyperproliferative effects in the rat colon (Sesink *et al*, 1999). To date, three cohort studies have reported on the association of heme iron intake and risk of colon or colorectal cancer (Lee *et al*, 2004; Larsson *et al*, 2005; Balder *et al*, 2006), and suggest a possible positive association of intake with risk, which may be enhanced in consumers of alcohol. However, there are inconsistencies in the association found in these studies by subsite (Lee *et al*, 2004; Larsson *et al*, 2005) and by sex (Balder *et al*, 2006), as well as with regard to the role of effect modification by alcohol consumption. We used data from a large cohort study of Canadian women to assess the risk of colorectal cancer in association with total iron and heme iron intake, as well as potential effect modification by alcohol consumption and hormone replacement use.

## METHODS AND MATERIALS

### Study population

Our investigation was conducted in the Canadian National Breast Screening Study (NBSS), a randomised controlled trial of screening for breast cancer, which has been described in detail elsewhere (Miller *et al*, 1992; Terry *et al*, 2002). In brief, 89 835 women aged 40–59 years were recruited from the general Canadian population between 1980 and 1985. On enrolment into the study, information was obtained from participants on demographic, hormonal, and reproductive characteristics using a self-administered lifestyle questionnaire. Starting in 1982, a self-administered food frequency questionnaire (FFQ) was distributed to all new attendees at all screening centres and to women returning to the screening centres for re-screening (Jain *et al*, 1982). The FFQ elicited information on usual portion size and consumption of 86 food items, and included photographs of portion sizes to assist respondents in quantifying intake. A total of 49 654 dietary questionnaires were returned and were available for analysis. We excluded 929 women with extreme energy intake values (at least three standard deviations above or below the mean value for natural log-transformed calories) and women whose body mass index (BMI) was <15 or >50 kg m^−2^ or who were missing information on BMI.

### Ascertainment of colorectal cancer and deaths

Incident cases of colorectal cancer and deaths from all causes were ascertained, respectively, by means of computerised record linkages to the Canadian Cancer Database and to the National Mortality Database. The linkages to the databases yielded data on cancer incidence and mortality up to 31 December 2000 for women in Ontario, 31 December 1998 for women in Quebec, and 31 December 1999 for women in other provinces. For the present analyses, study participants were considered at risk from their date of enrolment until the date of diagnosis of their colorectal cancer, termination of follow-up (the date to which cancer incidence data were available for women in the corresponding province), or death, whichever occurred first. During an average of 16.4 years (786 588 person-years) of follow-up of the dietary cohort, we identified 617 incident colorectal cancer cases.

### Computation of heme iron

Data from the FFQ were used to calculate total dietary iron intake using a database described elsewhere (Jain *et al*, 1982). The values for iron intake presented here are for dietary sources alone, because data on iron supplements were not collected. Total intake of meat iron was calculated from the reported intake of 22 meat items and two mixed dishes containing meat. Heme iron intake was computed by two methods, using different proportions for heme iron from different types of meat: 69% for beef; 39% for pork, ham, bacon, pork-based luncheon meats, and veal; 26% for chicken and fish; 21% for liver, according to Balder *et al* (2006), and, alternatively, using 40% as the average proportion of heme iron in all meats, according to Lee *et al* (2004). Results were similar for both methods, and we present data using the first approach. Total iron and heme iron intake were calorie-adjusted using the residuals method (Willett and Stampfer, 1986). In addition, we assessed risk in association with meat iron intake, red meat intake, and non-heme iron intake.

### Statistical analysis

After exclusion of the 18 women with prevalent colorectal cancer at baseline, and of those for the reasons described earlier, a total of 48 666 women were available for analysis, among whom there were 617 incident colorectal cancer cases. Cox proportional hazards models (using age as the time scale) were used to estimate hazard ratios (HR) and 95% confidence intervals (CI) for the association between iron intake and colorectal cancer risk. Quintiles of iron-related variables were derived based on their distribution in the total population. All multivariate models included the following covariates: age (time to event variable); BMI (kg m^−2^) (quintiles); menopausal status (pre-, peri-, and postmenopausal); oral contraceptive use (ever/never); hormone replacement use (ever/never); dietary intakes of fat, fiber, folic acid, total calories (all continuous); pack-years of smoking (never+five levels); alcohol intake (never drinker, >0 to <5 g day^−1^, 5 to <10 g day^−1^, 10 to <20 g day^−1^, 20 to <30 g day^−1^, 30 to <40 g day^−1^, and 40+g day^−1^); education (three levels); physical activity (none, moderate, vigorous, and missing). Addition of indicator variables for screening centre (1–15) and randomisation group in the original clinical trial (intervention/usual care) did not affect the risk estimates, and therefore these variables were omitted. Because an effect of iron or heme iron intake could be confounded by intake of other dietary constituents with which they are correlated, we included important food items (red meat, all meat, vegetables, and raw vegetables) in additional models. Inclusion of these variables did not affect the risk estimates for iron or heme iron, and as a result they are not included in the final models. We analysed the association of iron-related variables with colorectal cancer overall and with cancers of the colon, proximal colon, distal colon, and rectum. To test for trends in risk with increasing levels of the exposures of interest, we assigned the median value for each quintile and then fitted the medians as a continuous variable in the risk models. We then evaluated the statistical significance of the corresponding coefficient using the Wald test (Rothman and Greenland, 1998). In additional analyses, we also treated iron-related variables as continuous variables. Since early symptoms of the disease might result in dietary change, we repeated the analyses for colorectal cancer, excluding cases diagnosed during the first 3 years of follow-up.

We further examined the association of the iron-related variables with colorectal cancer risk within strata of potential effect modifiers: alcohol consumption (non-drinker; 1 to <20; and 20+ g day^−1^) and hormone replacement therapy (HRT) use (ever/never). Tests for interaction were based on the likelihood ratio tests comparing models with and without the product terms representing the variables of interest. All statistical significance tests were two-sided. All analyses were performed using SAS version 9 (SAS Institute Cary, NC, USA).

## RESULTS

Cases tended to be older than non-cases, were more likely to be postmenopausal, and were less likely to have used oral contraceptives (Table 1). Cases and non-cases were similar in terms of BMI, menstrual and reproductive variables, smoking, alcohol consumption, and hormone replacement use.

In multivariate models adjusting for potential confounding variables, quintiles of total iron intake, heme iron intake, and iron intake from meat showed no association with colon cancer, rectal, or colorectal cancer (Table 2). Intake of red meat was associated with increased risk of rectal cancer (HR for highest *vs* lowest quintile 1.95, 95% CI: 1.21–3.16, *P*-value for trend 0.008) but not with risk of colon cancer or all colorectal cancer. No associations or trends were seen when iron-related variables were treated as continuous variables (data not shown). The same variables also showed no associations with cancer of the proximal colon (218 cases) or distal colon (164 cases) (data not shown). When cases diagnosed during the first 3 years of follow-up were excluded from the analysis of colorectal cancer, the results were unchanged.

Total dietary iron intake was significantly correlated with intake of vegetables (*r*=0.49, *P*<0.0001), raw vegetables (*r*=0.26, *P*<0.0001), red meat (*r*=0.30, *P*<0.0001), and intake of all meats (*r*=0.50, *P*<0.0001). Intake of heme iron was inversely correlated with vegetable intake (*r*=−0.06) and positively correlated with intakes of red meat (*r*=0.51, *P*<0.0001) and all meats (*r*=0.74, *P*<0.0001). However, these foods were not associated with colorectal cancer risk, and their inclusion in the models did not alter the risk estimates for iron and heme iron.

There was no association between heme iron intake and colorectal cancer risk within strata of alcohol intake or hormone replacement use (Table 3). The results for total iron intake, meat iron intake, red meat intake, and non-heme iron intake were similar (data not shown). Furthermore, there were no significant interactions between heme iron intake and alcohol consumption or HRT use (Table 3) or of other iron-related variables with these potential effect modifiers.

## DISCUSSION

The present analysis showed no association between dietary intake of iron, heme iron, iron from meat sources, or non-heme iron and risk of cancer of the colon, rectum, or proximal or distal colon. Red meat intake was associated with increased risk of cancer of the rectum, with evidence of a dose–response relationship. Previous studies are suggestive of an association of red meat intake with colorectal cancer, but the evidence is weak and inconsistent (Potter, 1999). While potentially reflecting a real association, it is also possible that the result for rectal cancer is a chance finding due to the large number of comparisons. We found no association of any of iron- or meat-related variables with colorectal cancer in women who consumed any amount of alcohol or who consumed 20+ g day^−1^, or in users of HRT.

Three previous studies that have assessed the association between heme iron intake and colon or colorectal cancer have yielded mixed results. In the Iowa Women's Health Cohort, Lee *et al* (2004) reported a positive association of heme iron with proximal, but not distal, colon cancer, but only when zinc intake was included in the model. The association was stronger in women who consumed alcohol and strongest in women consuming 10+ g day^−1^ of alcohol. Using data from the Swedish Mammography Cohort, Larsson *et al* (2005) noted a statistically significant positive association between heme iron intake and colon cancer (principally distal colon cancer) in women who drank 20+ g week^−1^, but not in women drinking <20 g week^−1^. Finally, in an analysis of the Netherlands Cohort Study, Balder *et al* (2006) found a positive association between heme iron intake and colon cancer risk in men, but not in women, when cases diagnosed in the first 2 years of follow-up were excluded, but they did not observe an association with rectal cancer. The authors noted an increasing trend in risk of colon cancer in men only with increasing levels of the heme/chlorophyll ratio. They also reported finding a ‘suggestion of an association’ of heme iron with colon and rectal cancer in women who drank >5 g day^−1^ of alcohol. The studies by Lee *et al* and Larsson *et al* used a fixed proportion of heme iron (40%) from all types of meat, whereas Balder *et al* used differing proportions for different types of meat. We found no association using either method for calculation of heme iron.

Several other studies have examined intake of iron, either from the diet alone or from both diet and supplements, and markers of body iron stores in relation to colorectal cancer risk. In a Finnish cohort study, Knekt *et al* (1994) reported that participants with transferrin saturation levels of greater than 60% had a relative risk for colorectal cancer of 3.04 (95% CI: 1.64–5.62). In an analysis of data from the National Health and Nutrition Examination Survey I and the National Health Evaluation Follow-up Study (Wurzelmann *et al*, 1996), participants in the highest quartile of dietary iron intake were at increased risk for colon, but not rectal, cancer: relative risk for males and females combined 3.35 (95% CI: 1.74–6.46). In the same study, elevated serum iron was associated with increased risk of distal colon cancer in women only (relative risk, 2.74; 95% CI: 0.82–9.20) and rectal cancer (relative risk, 7.31; 95% CI: 1.13–47.2), whereas transferrin saturation levels and total iron binding capacity (TIBC) were not associated with colorectal cancer. In a nested case–control study carried out within a cohort of New York women (Kato *et al*, 1999), no overall associations were seen for levels of serum iron, ferritin, TIBC, transferrin saturation, or for iron intake (diet+supplements) with colorectal cancer. However, there was a significant trend over increasing quartiles of iron intake for cancer of the proximal colon (odds ratio for extreme quartiles 3.29; 95% CI: 0.7–14.6; *P* for trend=0.04).

Our study has a number of strengths. The large sample size made it possible to examine subsites within the colorectum. Follow-up of the population is virtually complete. We used two different approaches to estimating heme iron intake, with similar results, and we considered a variety of potential confounding variables as well as possible effect modification due to alcohol consumption and hormone replacement use. However, a number of limitations should also be pointed out. No information was available for iron derived from nutritional supplements, which, in a cohort study from New York (Kato *et al*, 1999), accounted for 38% of total iron intake. Additionally, zinc intake, which in two studies (Lee *et al*, 2004; Larsson *et al*, 2005) was inversely associated with risk of colon cancer, was not estimated from the original dietary database used in this study. Furthermore, only baseline questionnaire information was available, so that it is possible that intake of nutrients, including iron, changed over the long follow-up period, leading to non-differential misclassification of exposure.

In summary, in this large prospective cohort study, we found no suggestion of an association of iron or heme iron intake assessed at baseline with risk of subsequent colorectal cancer or with risk of cancer at subsites within the colorectum. Furthermore, there was no evidence of effect modification by alcohol consumption or hormone replacement use. To determine whether iron intake contributes to the development of colorectal cancer, future studies should improve on the assessment of iron intake by using repeat measurements and obtaining information on use of iron-containing supplements.

## Figures and Tables

**Table 1:** Baseline characteristics of the study population by outcome

**Factor**	**Incident colorectal cancer cases (*n*=617)**	**Non-cases (*n*=48 049)**
Mean age at baseline (years)	51.1 (5.4)	48.5 (5.6)
Mean BMI (kg m^−2^)^a^	24.8 (4.2)	24.8 (4.2)
Mean age at first live birth^b^	24.5 (4.1)	24.3 (4.8)
		
*Parity* (%)		
Nulliparous	14.6	14.9
1–2	30.8	35.8
3–4	42.9	38.6
5+	11.7	10.7
Postmenopausal (%)	53.0	37.2
		
*Cigarette smoking*		
Never	50.2	53.3
Former	29.2	27.3
Current	20.6	19.3
Alcohol intake (% drinking 10+ g day^−1^ of alcohol)	29.0	26.4
Oral contraceptive use (% ever)	48.0	59.5
HRT use (% ever)^c^	46.5	47.8

a BMI, body mass index.

b Among parous women.

c HRT, hormone replacement therapy; results among postmenopausal women only.

**Table 2:** Multivariate-adjusted HR and 95% CI for the association of intake of iron-related variables with risk of incident cancer of the colon, rectum, and colorectum

	**Multivariate HR^a^ (95% CI)**		
	**Colon cancer**	**Rectal cancer**	**Total colorectal cancer**
**Factor**	**(*n* cases=428)**	**(*n* cases=195)**	**(*n* cases=617^b^)**
*Total iron (mg day* ^−1^ *)*			
<11.90	1.00 (reference)	1.00 (reference)	1.00 (reference)
11.90 to <12.90	1.12 (0.82–1.54)	1.12 (0.71–1.78)	1.11 (0.86–1.44)
12.90 to <13.82	1.10 (0.81–1.52)	0.90 (0.55–1.48)	1.04 (0.80–1.36)
13.82 to <14.99	0.97 (0.69–1.36)	1.19 (0.74–1.92)	1.01 (0.77–1.34)
⩾14.99	1.07 (0.75–1.53)	1.12 (0.67–1.88)	1.07 (0.80–1.43)
*P* for trend	0.96	0.62	0.94
			
*Heme iron (mg day* ^−1^ *)*			
<1.58	1.00 (reference)	1.00 (reference)	1.00 (reference)
1.58 to <1.99	0.88 (0.64–1.19)	1.33 (0.82–2.15)	0.98 (0.75–1.27)
1.99 to <2.40	0.96 (0.71–1.30)	1.64 (1.03–2.62)	1.13 (0.87–1.45)
2.40 to <2.95	0.80 (0.57–1.12)	1.05 (0.62–1.78)	0.87 (0.65–1.15)
>2.95	0.99 (0.70–1.40)	1.27 (0.74–2.19)	1.06 (0.80–1.42)
*P* for trend	0.99	0.71	0.99
			
*Meat iron (mg day* ^−1^ *)*			
<3.40	1.00 (reference)	1.00 (reference)	1.00 (reference)
3.40 to <4.23	1.02 (0.75–1.38)	0.96 (0.60–1.54)	1.00 (0.77–1.29)
4.23 to <5.02	0.94 (0.69–1.29)	1.39 (0.89–2.17)	1.06 (0.82–1.37)
5.02 to <6.11	0.86 (0.62–1.20)	0.76 (0.45–1.28)	0.84 (0.63–1.11)
⩾6.11	1.06 (0.75–1.49)	1.06 (0.64–1.75)	1.06 (0.80–1.41)
*P* for trend	0.87	0.88	0.84
			
*Red meat (g day* ^−1^ *)*			
<14.25	1.00 (reference)	1.00 (reference)	1.00 (reference)
14.25 to <21.02	1.06 (0.79–1.42)	1.25 (0.75–2.08)	1.10 (0.85–1.42)
21.02 to <28.74	0.97 (0.72–1.32)	1.79 (1.11–2.88)	1.17 (0.90–1.50)
28.74 to 40.30	0.84 (0.61–1.15)	1.42 (0.85–2.35)	0.97 (0.74–1.27)
40.30+	0.88 (0.64–1.21)	1.95 (1.21–3.16)	1.12 (0.86–1.46)
*P* for trend	0.16	0.008	0.66

CI, confidence intervals; HR, hazard ratios.

a Adjusted for age (time to event variable); body mass index (kg m^−2^) (quintiles); menopausal status (pre-, peri-, and postmenopausal); oral contraceptive use (ever/never); hormone replacement use (ever/never); dietary intakes of fat, fibre, folic acid, total calories (continuous); pack-years of smoking (never+five levels); alcohol intake (never drinker, >0 to <5, 5 to <10, 10 to <20, 20 to <30, 30 to <40, and 40+ g day^−1^); education (three levels); physical activity (none, moderate, vigorous, and missing).

b Six women had a diagnosis of both colon and rectal cancer and were included in the analyses for both the colon and the rectum.

**Table 3:** Multivariate-adjusted HR and 95% CI for the association between heme iron intake and colorectal cancer, stratified by potential effect modifiers

**Stratification variables**	**HR^a^ (95% CI)**	**HR^a^ (95% CI)**	**HR^a^ (95% CI)**
Alcohol intake	Non-drinker (*n* cases=142)	Drinker 1 to <20 g day^−1b^ (*n* cases=364)	Drinker 20+ g day^−1c^ (*n* cases=83)
*Heme iron intake*			
<1.58	1.00 (reference)	1.00 (reference)	1.00 (reference)
1.58 to <1.99	0.77 (0.38–1.56)	0.99 (0.71–1.39)	0.88 (0.42–1.84)
1.99 to <2.40	0.72 (0.35–1.47)	1.19 (0.87–1.65)	0.76 (0.33–1.72)
2.40 to <2.95	1.11 (0.58–2.14)	0.74 (0.51–1.07)	0.78 (0.31–1.94)
>2.95	1.07 (0.54–2.11)	0.97 (0.67–1.41)	1.21 (0.46–3.15)
*P* for trend	0.54	0.43	0.98
			
HRT^d^	Never	Ever	
	(*n* cases=175)	(*n* cases=152)^e^	
			
*Heme iron intake*			
<1.58	1.00 (reference)	1.00 (reference)	
1.58 to <1.99	0.96 (0.60–1.54)	1.02 (0.60–1.74)	
1.99 to <2.40	1.15 (0.72–1.83)	1.42 (0.86–2.34)	
2.40 to <2.95	0.84 (0.50–1.40)	1.14 (0.65–1.98)	
>2.95	1.02 (0.60–1.74)	1.40 (0.79–2.47)	
*P* for trend	0.89	0.25	

CI, confidence intervals; HR, hazard ratios; HRT, hormone replacement therapy.

a Adjusted for age (time to event variable) and all except the stratification variable: body mass index (kg m^−2^) (quintiles); menopausal status (pre-, peri-, and postmenopausal); oral contraceptive use (ever/never); hormone replacement use (ever/never); dietary intakes of fat, fibre, folic acid, total calories (continuous); pack-years of smoking (never+five levels); alcohol intake (continuous); and education (three levels).

b *P* for test for interaction between this level of drinking (*vs* never drinking) and heme iron intake=0.69.

c *P* for test for interaction between this level of drinking (*vs* never drinking) and heme iron intake=016.

d Restricted to postmenopausal women.

e *P* for test for interaction between HRT and heme iron intake=0.49.
