# A cohort study of dietary iron and heme iron intake and risk of colorectal cancer in women

**DOI:** 10.1038/sj.bjc.6604107

**Published:** 2007-11-27

**Authors:** G C Kabat, A B Miller, M Jain, T E Rohan

**Correction to:** *British Journal of Cancer* (2007) **97**, 118–122. doi:10.1038/sj.bjc.6603837

Owing to an error by the author in the algorithm for computing red meat intake, the values for the cut points and the hazard ratios and confidence intervals in the bottom-most panel of Table 2 were incorrect.

The correct values are given below.

The authors would like to point out that there is still a positive association of red meat intake with rectal cancer but not with colon cancer or all colorectal cancers. Therefore, the correction does not affect the interpretation presented in the paper.

## Figures and Tables

**Table 2:** Multivariate-adjusted HR and 95% CI for the association of intake of iron-related variables with risk of incident cancer of the colon, rectum, and Colorectum

	**Multivariate HR^a^ (95% CI)**		
**Factor**	**Colon cancer (*n* =428)**	**Rectal cancer (*n* =195)**	**Total colorectal cancer (*n* =617^b^)**
*Red meat (g day* ^*−1*^ *)*			
<48.49	1.00 (reference)	1.00 (reference)	1.00 (reference)
48.49 to <66.33	0.90 (0.66–1.22)	1.70 (1.04–2.79)	1.08 (0.84–1.40)
66.33 to <83.47	1.00 (0.74–1.36)	1.61 (0.97–2.67)	1.15 (0.88–1.49)
83.47 to <108.99	0.79 (0.57–1.10)	1.41 (0.82–2.40)	0.94 (0.71–1.25)
108.99+	0.98 (0.70–1.36)	2.09 (1.24–3.53)	1.23 (0.93–1.63)
*P* for trend	0.62	0.04	0.39

CI=confidence intervals; HR=hazard ratios.

a Adjusted for age (time to event variable); body mass index (kg m^−2^) (quintiles); menopausal status (pre-, peri-, and postmenopausal); oral contraceptive use (ever/never); hormone replacement use (ever/never); dietary intakes of fat, fibre, folic acid, total calories (continuous); pack-years of smoking (never + five levels); alcohol intake (never drinker, >0 to <5, 5 to <10, 10 to <20, 20 to <30, 30 to <40, and 40+ g day^−1^); education (three levels); physical activity (none, moderate, vigorous, and missing).

b Six women had a diagnosis of both colon and rectal cancer and were included in the analyses for both the colon and the rectum.

